# Cardiovascular complications in chronic active Epstein-Barr virus infection: a narrative review

**DOI:** 10.3389/fcvm.2026.1651391

**Published:** 2026-03-18

**Authors:** Jing Xu, Yunfeng Wei, Lin Shi

**Affiliations:** 1 Department of Ultrasound, Mianyang Central Hospital, University of Electronic Science and Technology of China, Mianyang, Sichuan, China; 2 Department of Medical Oncology, Sichuan Clinical Research Center for Cancer, Sichuan Cancer Hospital & Institute, Sichuan Cancer Center, Affiliated Cancer Hospital of University of Electronic Science and Technology of China, Chengdu, China

**Keywords:** cardiogram, cardiovascular complication, chronic active Epstein–Barr virus, coronary artery lesions, pulmonary arterial hypertension

## Abstract

**Background:**

Cardiovascular involvement in chronic active Epstein–Barr virus (CAEBV) infection represents an uncommon yet severe spectrum of complications, including coronary artery lesions, arrhythmias, and pulmonary arterial hypertension. Reliable large-scale data for the management of CAEBV-associated cardiovascular damage are currently lacking. In this literature review, we aimed to summarize the clinical characteristics, pathological features, diagnostic approaches, and treatment strategies for CAEBV-related cardiac damage. This summary may enhance the awareness of CAEBV-associated cardiovascular complications and provide valuable insights for their clinical management. In this narrative review, we aimed to summarize the clinical characteristics, pathological features, diagnostic approaches, and treatment strategies for CAEBV-related cardiac damage.

**Methods:**

PubMed, Embase, Web of Science, CNKI, CQVIP, and Wanfang Data databases were searched as part of this narrative review to collect data on CAEBV infection with cardiovascular involvement and to summarize its characteristics, pathological features, diagnostic approaches, and treatment strategies.

**Results:**

We identified 32 articles reporting CAEBV-associated cardiovascular complications involving 57 patients with a median age of 11 years (range: 1–69 years). Among these patients, 19 (33.3%) were adults and 38 (66.7%) were children; 24 (42.1%) were male and 33 (57.9%) were female. Coronary artery lesions, electrocardiographic abnormalities, valvular regurgitation, and pericardial lesions were more frequent in children, whereas heart failure and pulmonary arterial hypertension were more prevalent in adults.

**Conclusions:**

Cardiovascular involvement in CAEBV infection is rare and presents substantial diagnostic and therapeutic challenges. The systematic evaluation of cardiovascular involvement is crucial for timely intervention and long-term surveillance. The poor prognosis of CAEBV-associated cardiovascular complications underscores the urgent need for further research to elucidate its pathogenic mechanisms and optimize therapeutic strategies.

## 1 Introduction

The Epstein–Barr virus (EBV), also known as human herpes virus 4, was discovered by Epstein and Barr in 1964. EBV is a ubiquitous pathogen that infects over 90% of the global population (1). In a subset of genetically susceptible individuals, persistent infection of T or NK cells following EBV exposure can lead to T or NK cell-dominant EBV-associated lymphoproliferative diseases such as chronic active Epstein–Barr virus (CAEBV) infection. CAEBV infections are predominantly reported in East Asia and Latin America, with T or NK cell involvement as the hallmark feature. In contrast, CAEBV infection has a lower incidence in Western countries, where B-cell infections are more prevalent (2, 3). Recent studies have detected EBV in the hematopoietic stem cells (HSCs) of patients with CAEBV infection. Further lineage-specific analyses indicate that EBV infection occurs not only in T cells, B cells, and NK cells, but also in differentiated myeloid lineages such as monocytes, neutrophils, megakaryocytes, and erythrocytes (4).

Currently, CAEBV is defined based on detailed diagnostic criteria (5). CAEBV infection is associated with life-threatening complications including infectious mononucleosis–like syndrome, virus-associated hemophagocytic syndrome, malignant lymphoma, disseminated intravascular coagulation, interstitial pneumonia, hepatic failure, and cardiovascular disease, which contribute to its high mortality rate.

Cardiovascular involvement in CAEBV infection presents a severe spectrum of complications, including coronary artery lesions (CALs), heart failure, pulmonary arterial hypertension (PAH), arrhythmias, and pericardial effusion. Studies have reported circulatory system complications in 9.8–17.9% of patients with CAEBV infection, with myocarditis and coronary artery aneurysms (CAAs) occurring in 6% and 9%, respectively (6–8). However, the absence of large-scale data and evidence-based guidelines poses substantial challenges in the management of these complications.

The pathogenic mechanisms underlying CAEBV-induced cardiovascular injury remain unclear but are broadly classified into direct and indirect pathways. Direct injury primarily involves infiltration and cytotoxic damage to the myocardial tissue, pericardium, and vasculature by EBV-infected T or NK cells during CAEBV infection, resulting in myocarditis, pericardial effusion, and arteritis (9). Infiltrating lymphocytes may penetrate the full myocardial thickness and epicardial adipose tissue, thereby inducing cardiomyocyte lysis and necrosis. Hasegawa et al. identified specific myocardial degeneration in patients with CAEBV infection, which is potentially linked to arrhythmogenesis (10). Vascular invasion presents as lymphocytic vasculitis, which is characterized by irregular mural thickening primarily due to intimal hyperplasia and moth-eaten destruction of the medial elastic lamina with multifocal fragmentation (11, 12).

Accumulating evidence has underscored the importance of immune-mediated indirect injury mechanisms, primarily centered on cytokine storms, immune cell infiltration, and endothelial dysfunction. EBV-encoded latent membrane protein 1 (LMP-1) upregulates vascular endothelial growth factor (VEGF) production, thereby increasing vascular permeability and promoting vascular wall degradation (13). In contrast, EBV-infected NK or T cells facilitate the secretion of adhesion molecules and cytokines, exacerbating vascular lesions (14). Furthermore, during the replicative cycle, EBV generates deoxyuridine triphosphatase (dUTPase), which elevates interleukin-6 (IL-6) levels, induces vascular endothelial injury, and leads to coronary artery abnormalities (15, 16). Additionally, activated innate immune and cardiac cells release cytokines, chemokines, interferons, and alarmins, leading to further activation and recruitment of innate immune cells, including mast cells, neutrophils, dendritic cells, monocytes, and macrophages, to the heart (17, 18). Antigen presentation subsequently activates adaptive immunity, particularly in T lymphocyte subsets. Excessive or persistent activation of these immune pathways is critical for the initiation and aggravation of chronic inflammation (13, 14, 18).

This review summarizes recent advancements in the manifestations, diagnosis, treatment, and prognosis of cardiovascular complications associated with CAEBV infection with the aim of enhancing clinical understanding and management.

## 2 Demographic characteristics of CAEBV infection patients with cardiovascular involvement

PubMed, Embase, Web of Science, CNKI, CQVIP, and Wanfang Data databases were searched to identify literature on CAEBV infection with cardiovascular involvement from inception to December 2024. After excluding duplicate articles, those with incomplete data, and those with unavailable full texts, 32 articles (10–12, 19–47) reporting CAEBV-associated cardiovascular complications remained: 19 from Japan (10–12, 20–22, 24–31, 35, 36, 38, 45, 46), 8 from China (19, 33, 34, 37, 39, 41, 42, 44), and 1 article each from Morocco (40), Germany (23), South Korea (32), Brazil (43), and India (47) (Table 1). Fifty-seven patients were included, consisting of 19 adults (33.3%) and 38 children (66.7%), with an overall median age of 11 years (range, 1–69 years). The median age of the pediatric patients was 6 years (range, 1–16 years), while that of the adults was 36 years (range, 19–69 years). Among the cohort, 24 (42.1%) were male and 33 (57.9%) were female. T-cell infection was identified in 33 patients (57.9%), followed by NK-cell infection in 9 patients (15.8%); in 15 patients (26.3%), it was not mentioned. For outcomes, 24 patients (42.1%) remained alive, whereas 30 patients (52.6%) died. Where reported, deaths were attributable to cardiovascular complications (e.g., advanced heart failure or sudden cardiac arrest), systemic CAEBV-related complications (e.g., hemophagocytic syndrome with multi-organ failure or severe infection/respiratory failure), or treatment-related causes (e.g., transplantation-related complications or lymphoma progression). In several cases, the cause of death was not specified in the original reports; these are labeled as NA in Supplementary Table.

**Table 1:** Literature review of the cardiovascular involvement in chronic active Epstein–Barr virus infection.

Year	Author	Country	Patient	Age (years)	Gender	Cell type	Cardiac complications	Outcomes
1989	Kobayushi et al. (20)	Japan	Pt.1	2	M	T	CAA	NA
1992	Yamada et al. (21)	Japan	Pt.2	23	M	T	PE	Dead
1993	Kikuta et al. (22)	Japan	Pt.3	2	M	NA	CAA, dilatation of Valsalva sinus	Dead
			Pt.4	6	F	NA	CAA, Pericarditis	Dead
			Pt.5	5	F	NA	CAA, dilatation of Valsalva sinus, Pericarditis	Dead
1996	Nakagawa et al. (11)	Japan	Pt.6	5	F	T	CAA, dilatation of Valsalva sinus, thoracic and abdominal aortic aneurysms	Dead
1998	Murakami et al. (12)	Japan	Pt.7	10	F	T	CAA, multiple arterial aneurysms (including bilateral common carotid and subclavian arteries, abdominal aorta and its major branches, and bilateral common iliac arteries)	Dead
2001	Hauptmann et al. (23)	Germany	Pt.8	28	M	T	Dilated cardiomyopathy, congestive heart failure (LVEF = 15%)	Dead
2006	Toubo et al. (24)	Japan	Pt.9	7	F	T	CAD, PE, tachycardia, prolonged PR interval	Dead
2006	Sato et al. (25)	Japan	Pt.10	11	F	NK	Dilatation of the Valsalva sinus, AR	Dead
2008	Takano et al. (26)	Japan	Pt.11	45	M	T	Myocarditis, Arrhythmia (AF and sporadic PVCs), heart failure	Dead
2009	Muneuchi et al. (27)	Japan	Pt.12	1	M	T	Complete AV block	Dead
			Pt.13	2	F	NK	CAL	Alive
2009	Muneuchi et al. (27)	Japan	Pt.14	3	F	T	Low LVEF, PE	Dead
			Pt.15	5	M	T	CAL	Dead
			Pt.16	6	M	T	CAL	Alive
			Pt.17	9	F	T	CAL	Dead
			Pt.18	11	F	NK	Sudden cardiac arrest	Dead
			Pt.19	14	F	T	Fatal PE	Dead
			Pt.20	16	F	T	Low LVEF, PE	Dead
2009	Hasegawa et al. (10)	Japan	Pt.21	12	M	T	CAD	Dead
2011	Hashimoto et al. (28)	Japan	Pt.22	45	M	NK	PAH	Dead
2014	Onishi et al. (29)	Japan	Pt.23	36	F	T	PAH, heart failure	Alive
2014	Nishimura et al. (30)	Japan	Pt.24	26	F	NA	CAA, RCA was occluded, bilateral vertebral artery aneurysms, left vertebral artery asymptomatic occlusion	NA
2015	Fukuda et al. (31)	Japan	Pt.25	6	M	NA	PAH, CAA, arrhythmia (junctional ectopic tachycardia), CRBBB	Dead
2015	Kim et al. (32)	Korea	Pt.26	5	M	T	CAD, calcification of aortic root	Dead
2016	Jiang et al. (33)	China	Pt.27	16	F	NA	CAA and stenoses, aortic aneurysms, myocardial infarction	NA
2019	Ba et al. (34)	China	Pt.28	9	M	NA	PAH, CAA, dilatation of the Valsalva sinus	Alive
2020	Akagi et al. (35)	Japan	Pt.29	44	M	NK	PAH	Dead
2020	Kang et al. (36)	Japan	Pt.30	42	M	NA	CAA, abdominal aortic aneurysm and bilateral common iliac artery (CIA) aneurysms	Alive
2020	Xiao et al. (37)	China	Pt.31	4	F	T	CAA, mitral and aortic valve insufficiency, thickened aortic wall, stenosis and dilatation of the abdominal aorta	Alive
2021	Sasagasako et al. (38)	Japan	Pt.32	36	F	T	CAA, myocardial infarction, heart failure, the left vertebral artery aneurysm was occluded by thrombus, frequent PVCs	Alive
			Pt.33	32	F	T	PAH	Alive
2021	Wei et al. (39)	China	Pt.34	10.5	F	7T,3NK	CAA, mild AR and MR, T-wave inversion	Alive
			Pt.35	6.3	M		CAA, prolonged PR interval	Alive
			Pt.36	12.2	M		CAA, mild MR, AR and TR, abnormal ST-T voltage, mild PE	Alive
			Pt.37	5.8	F		CAA	Alive
			Pt.38	14.3	M		CAA, mild TR, prolonged PR interval, mild PE	Alive
			Pt.39	3.2	M		CAA, mild PE	Alive
2021	Wei et al. (39)	China	Pt.40	7.6	M	7T,3NK	CAA, mild TR, prolonged PR interval	Dead
			Pt.41	4.3	F		CAA, mild MR and AR, mild PE	Alive
			Pt.42	2.8	F		CAA	Alive
			Pt.43	5	M		CAA	Alive
2021	Jamal et al. (40)	Morocco	Pt.44	22	M	T	CAA, multiple arterial aneurysms (hepatic artery, gastroduodenal artery, splenic artery, superior mesenteric artery and common iliac arteries)	Dead
2022	Li et al. (41)	China	Pt.45	5	F	NA	CAA, Sinus of Valsalva Aneurysms, multiple arterial aneurysms, moderate MR and TR, mild AR, second-degree atrioventricular block.	Dead
2022	Pi et al. (42)	China	Pt.46	36	F	T	Massive PE, ectopic atrial rhythm, atrial tachycardia and first-degree atrioventricular block	Alive
2022	Paula et al. (43)	Brazil	Pt.47	19	M	NA	CAA, mild PE, multiple arterial aneurysms (splenic, superior mesenteric, common hepatic, left gastric, celiac trunk, bilateral intercostal, subclavian, mammary, carotid and vertebrobasilar system, thoracic and abdominal aorta)	Dead
2022	Teng et al. (43, 44)	China	Pt.48	9	F	T	CAA	Alive
2023	Misaki et al. (45)	Japan	Pt.49	23	F	T	PAH	Alive
			Pt.50	41	F	NK	PAH	Dead
			Pt.51	69	F	NA	PAH	Dead
2023	Misaki et al. (45)	Japan	Pt.52	30	F	T	PAH	Dead
2023	Qian et al. (19)	China	Pt.53	2	F	NA	CAA, CRBBB	Alive
			Pt.54	13	M	T	CAA	Alive
			Pt.55	3	F	NA	CAA	Alive
2024	Iwata et al. (46)	Japan	Pt.56	24	F	NA	CAA and chronic total occlusion	Alive
2024	Raghuram et al. (47)	India	Pt.57	37	F	NA	Left ventricular apical pseudoaneurysm, moderate AR, moderate PE, stenosis of the proximal segments of bilateral subclavian arteries and infrarenal abdominal aorta. Dilatations of the distal left subclavian artery and bilateral common iliac arteries, sinus tachycardia, normal QRS axis, T wave inversion noted in leads II, III, aVF and V2-V6.	Dead

F, female; M, male; PE, pericardial effusion; LVEF, left ventricular ejection fraction; CAD, coronary artery dilatation; CAA, coronary artery aneurysm; CAL, coronary artery lesion; PAH, pulmonary arterial hypertension; AR, aortic regurgitation; MR, mitral regurgitation; TR, tricuspid regurgitation; RCA, right coronary artery; CRBBB, complete right bundle branch block; AF, atrial fibrillation; PVCs, premature ventricular contractions.

Treatment reporting across the included cases is summarized in Supplementary Table. Reported therapies included antivirals (e.g., acyclovir or ganciclovir), immunosuppression (most commonly corticosteroids, sometimes combined with cyclosporine), chemotherapy/HLH-directed regimens (e.g., etoposide-based therapy), allogeneic hematopoietic stem cell transplantation (HSCT), and cardiovascular-targeted management (e.g., anticoagulation/antiplatelet therapy for coronary artery lesions, pericardiocentesis for tamponade, and pulmonary vasodilators for PAH when reported). When treatment details were not available in the original reports, this is explicitly indicated as NA in the tables.

**Figure 1:**
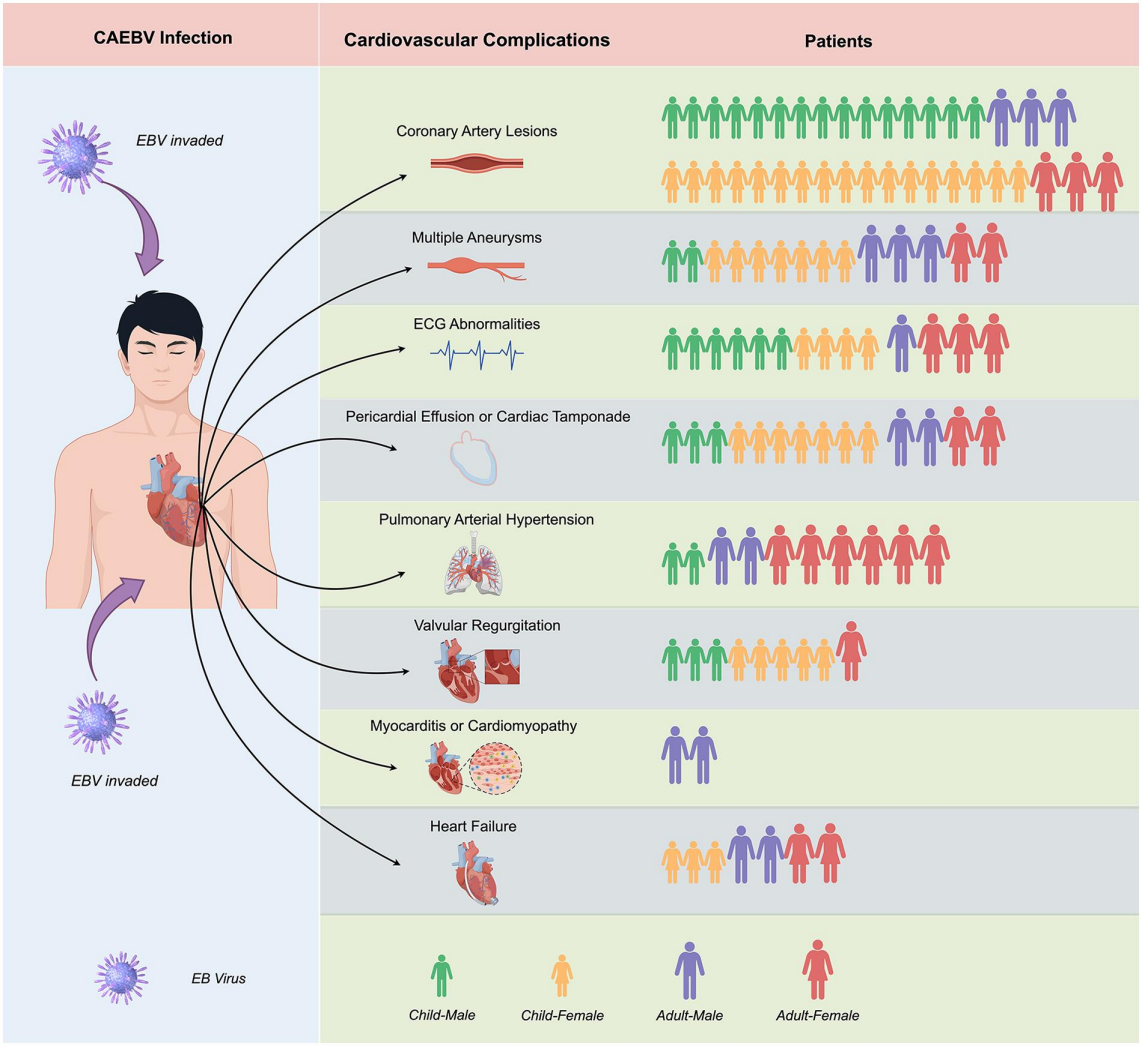
Demographic characteristics of chronic active Epstein–Barr virus (CAEBV) infection with different cardiovascular complications. Each illustrated person icon represents one patient; green (male) and orange (female) icons indicate children, while purple (male) and red (female) icons indicate adults. Coronary artery lesions, multiple aneurysms, electrocardiogram (ECG) abnormalities, pericardial effusion (including cardiac tamponade), and valvular regurgitation were more frequently observed in children, whereas pulmonary arterial hypertension (PAH), myocarditis/cardiomyopathy, and heart failure were more prevalent in adults. Data are based on the 57 reported CAEBV cases with cardiovascular involvement included in this narrative review. *This figure was drawn on the Figdraw website* (https://www.figdraw.com).

The characteristics of the CAEBV-associated cardiovascular complications are summarized in Table 2. Consistent with previous reports, CALs, valvular regurgitation, multiple vascular lesions, pericardial effusion, and electrocardiographic abnormalities were observed more frequently in children. In contrast, heart failure was more prevalent in adults. Additionally, our findings indicated that adults have a higher likelihood of developing concurrent PAH (Figure 1).

**Table 2:** The clinical characteristics of CAEBV-associated cardiovascular complications.

Complication	Articles (No.)	Patients (No.)	Child (No.)	Adult (No.)	Male (No.)	Female (No.)	Age (years)			Country (Articles/Patients) (No.)						
							All	Child	Adult	Japan	China	Korea	Germany	Morocco	Brazil	India
Total	32	57	38	19	24	33	11 (1–69)	6 (1–16)	36 (19–69)	19/33	8/19	1/1	1/1	1/1	1/1	1/1
CALs	22	38	32	6	18	20	6 (2–42)	6 (2–16)	25 (19–42)	12/17	7/18	1/1		1/1	1/1	
Multiple aneurysms	13	14	9	5	5	9	10 (2–42)	5 (2–16)	36 (19–42)	6/7	4/4			1/1	1/1	1/1
ECG abnormalities	10	14	10	4	7	7	9 (1–45)	7 (1–14)	37 (36–45)	5/5	4/8					1/1
PE	8	14	10	4	5	9	13 (3–37)	6 (3–16)	29 (19–37)	4/7	2/5				1/1	1/1
PAH	7	10	2	8	4	6	32 (6–69)	8 (6–9）	38 (23–69)	6/9	1/1					
Valvular regurgitation	5	9	8	1	3	6	10 (4–37)	7 (4–14)	37	1/1	3/7					1/1
Myocarditis/cardiomyopathy	2	2	0	2	2	0	37 (28–48)	/	37 (28–48)	1/1			1/1			
Heart failure	5	7	3	4	2	5	28 (3–45)	11 (3–16)	36 (28–45)	4/6			1/1			

CAEBV, chronic active Epstein–Barr virus; PE, pericardial effusion; CALs, coronary artery lesions; PAH, pulmonary arterial hypertension.

## 3 Different cardiovascular manifestations and diagnosis in patients with CAEBV infection

### 3.1 Coronary artery lesions

CALs associated with CAEBV infection, which primarily manifest as coronary artery dilatation (CAD) or CAAs, are diagnosed by measuring the diameter of the coronary lumen using echocardiography or coronary angiography. CAAs are typically defined as localized dilatations in which the diameter of the aneurysmal segment exceeds 1.5 times that of the adjacent normal artery (48). In recent years, the *Z*-score, which is calculated based on body surface area, has become the predominant evaluation metric for CALs, particularly in pediatric patients, as it demonstrates broader applicability than absolute coronary diameter measurements. Based on the *Z*-score, lesions were classified into 5 categories: normal, CAD-only, small CAA, medium CAA, and large or giant CAA (49). Although coronary atherosclerosis is the most common cause of coronary abnormalities in adults, pediatric cases involve both congenital and acquired etiologies, with Kawasaki disease (KD) being the predominant acquired factor (48). Literature reports indicate an 8.9% incidence of CALs in patients with CAEBV infection (39). Previous studies have paid limited attention to CAEBV-associated coronary lesions and have often overlooked this type of vascular pathology.

There were 22 articles (10–12, 19, 20, 22, 24, 27, 30–34, 36–41, 43, 44, 46) with 38 patients reporting CAEBV-associated CALs, 12 of which were Japanese (17 patients), 7 were Chinese (18 patients), and 1 article each for Morocco, Brazil, and Korea. Among these patients, 32 (84.2%) were children and 6 (15.8%) were adults, with 18 males (47.4%) and 20 females (52.6%). The median age was 6 years (range, 2–42 years), with the pediatric and adult subgroups showing median ages of 6 years (range, 2–16 years) and 25 years (range, 19–42 years), respectively. CALs frequently coexisted with other cardiovascular abnormalities: 12 patients (31.6%) presented with isolated CALs, whereas 26 (68.4%) exhibited coexisting cardiovascular damage. Of the 26 patients, 10 had additional vascular lesions such as multiple aneurysms in the aorta and branch vessels, 10 showed electrocardiographic (ECG) abnormalities, 8 had pericardial involvement, 7 demonstrated valvular regurgitation, 5 exhibited a sinus of Valsalva aneurysm, 4 progressed to myocardial infarction or heart failure, and 2 developed PAH.

Patients with CAEBV-associated CALs may exhibit diverse manifestations depending on the severity of the CALs and concurrent comorbidities, ranging from chest pain, congestive heart failure, acute coronary syndrome, and sudden death to being entirely asymptomatic. An elevated EBV viral load, fever, and cytopenia have been proposed as potential contributors to cardiovascular complications (27). The Coronary Artery Aneurysm Registry, the largest multicenter registry, reported a mortality rate of 15.3% and major adverse cardiac event rate of 31% in adults (50). However, no specific prognostic data have been reported for CAEBV infections with CALs. In our review, 52.6% of patients with CAEBV-associated CALs survived after treatment. Wei et al. reported that 30% of patients' coronary arteries with CALs returned to normal, and no patient died of CALs-related complications (39). Additionally, patients with CAEBV-associated CAAs with chronic total occlusion that underwent coronary artery bypass grafting (CABG) exhibited restoration of normal coronary circulation, as reported in the literature (46).

Diagnosing CALs is not inherently challenging; however, confirming whether CALs are attributable to CAEBV infection or KD, particularly incomplete KD, remains a diagnostically complex task. Kikuta et al. reported a high positivity rate for EBV-DNA polymerase chain reaction (PCR) testing in peripheral blood samples from patients with KD, suggesting that EBV infection may be a potential etiological contributor to KD (51). This complicates the differentiation between KD and CAEBV infection in patients with CALs. Definitive determination of the underlying cause of coronary damage is impossible unless coronary tissue is available for EBV DNA testing, a procedure that is rarely feasible in clinical practice. Because such biopsies are seldom performed in living patients owing to their invasiveness and risk, current diagnostic practices remain insufficient to definitively confirm whether lesions result from CAEBV infection or KD. In the absence of histopathological evidence, clinical symptoms and follow-ups may provide partial diagnostic guidance. Typically, KD coronary manifestations appear 7–10 days after disease onset, whereas in CAEBV infections, such manifestations may develop over time. The predominance of neutrophil activation in KD vs. lymphocyte activation in CAEBV infection may aid in the differentiation between these 2 conditions. Notably, many patients with KD lack the typical symptoms and only present with CAAs. Given the high risk of aneurysm rupture, intravenous immunoglobulin (IVIG) is administered when incomplete KD is suspected to prevent CAL progression (9). Further diagnostic evaluation should be performed based on persistent clinical manifestations after IVIG treatment.

### 3.2 Multiple aneurysms

Patients with CAEBV infection may develop vascular involvement that extends beyond CALs. These changes are characterized by dilated or stenotic alterations in any segment of the aorta and its branch arteries, including the subclavian, vertebral, superior mesenteric, and common iliac arteries. Studies have reported the occurrence of multiple aneurysms in patients with CAEBV infection, not only in the coronary arteries, but also in the splenic, superior mesenteric, common hepatic, left gastric, celiac trunk, bilateral intercostal, subclavian, carotid, vertebrobasilar systems, and thoracoabdominal aorta (40, 43). Clinical presentations vary according to lesion severity, ranging from asymptomatic to life-threatening complications, such as intracranial hemorrhage due to vascular rupture, acute cardiac tamponade resulting from aneurysm rupture, or cardiocerebrovascular arrest (40).

We identified 13 articles (11, 12, 22, 25, 30, 33, 34, 36, 37, 40, 41, 43, 47) reporting multiple CAEBV-associated aneurysms involving 14 patients. These 13 articles included 6 Japanese studies (7 patients), 4 Chinese studies (4 patients), and a single case each from Morocco, Brazil, and India. The cohort consisted of 9 pediatric patients (64.3%) and 5 adult patients (35.7%), with a male-to-female ratio of 5:9 (35.7% vs. 64.3%). The overall median age was 10 years (range, 2–42 years), stratified as 5 years (range, 2–16 years) for the pediatric subgroup and 36 years (range, 19–42 years) for the adult subgroup.

Distinguishing CAEBV-associated vasculopathy from Takayasu arteritis (TA) is critical despite overlapping features such as aortic branch vessel stenosis, occlusion, or dilatation. TA, a type of vasculitis potentially associated with genetic or immune dysregulation, is characterized by distinct histopathological features, including granulomatous inflammation of the arterial walls in the active phase, which may progress to panarteritis with minimal inflammation and adventitial fibrosis in the chronic stages (52, 53). TA predominantly affects young women aged 20–40 years and presents with pulse deficits, blood pressure discrepancies, vascular bruits, and elevated inflammatory markers such as erythrocyte sedimentation rate (ESR) and C-reactive protein (CRP) level, but usually lacks autoantibodies. In contrast, CAEBV-related vasculopathy results from persistent EBV infection and is characterized by systemic lymphocytic infiltration. Histopathological hallmarks include perivascular atypical lymphocyte infiltration, destruction of the medial elastic lamina at vascular bifurcations with a moth-eaten appearance, and EBV-encoded RNA (EBER) positivity along with elevated EBV DNA levels in affected vessels (11). CAEBV-associated vascular lesions exhibit a broader age distribution, primarily affecting pediatric and adolescent populations, with the involvement of medium or small arteries. These lesions are often accompanied by systemic manifestations, such as fever, hepatosplenomegaly, and lymphadenopathy. Timely differentiation is imperative because of the distinct therapeutic approaches and prognoses associated with these entities.

### 3.3 Electrocardiographic abnormalities

A subset of patients with CAEBV infection may exhibit ECG abnormalities including tachycardia, atrial fibrillation, atrioventricular block, junctional escape beats, premature contractions, and ST-T changes. These abnormalities may result from the direct viral infiltration of cardiomyocytes or conduction pathways, leading to myocardial degeneration or conduction disturbances. Autopsy has revealed EBER-positive cells surrounding the degenerated myocardium in CAEBV-infected patients, with instances of transient ventricular fibrillation (10). Case reports have further associated EBV-related myocarditis with atrioventricular block, suggesting that persistent EBV infection may induce cardiomyocyte degeneration, thereby triggering arrhythmias or acute circulatory failure (54). Alternatively, ECG abnormalities may secondarily reflect heart structural remodeling, as observed in CAEBV-associated PAH. Severe PAH often leads to right ventricular enlargement and septal shift, which manifests as right axis deviation and complete right bundle branch block (31).

We retrieved 10 articles with 14 patients (19, 24, 26, 27, 31, 38, 39, 41, 42, 47) documenting CAEBV-associated ECG abnormalities, 5 of which were Japanese (5 patients), 4 were Chinese (8 patients), and 1 was Indian. Of the included patients, 10 were pediatric (71.4%) and 4 were adults (28.6%). There were 7 males (50.0%) and 7 females (50.0%). The overall median age was 9 years (range, 1–45 years), with a median of 7 years (range, 1–14 years) in the pediatric subgroup and 37 years (range, 36–45 years) in the adult subgroup.

CAEBV-related ECG abnormalities may present asymptomatically or with symptoms, such as chest tightness, palpitations, or syncope. ECG exhibits nearly 100% diagnostic accuracy in detecting CAEBV-associated arrhythmias, providing direct visualization of the heart rate, rhythm patterns, and specific arrhythmia subtypes. This modality facilitates timely therapeutic decision-making and plays a critical role in longitudinal monitoring, thereby justifying routine ECG evaluation in all patients with CAEBV infection.

### 3.4 Pericardial effusion or cardiac tamponade

Pericardial effusion or cardiac tamponade may occur in patients with CAEBV infection, either as an isolated condition or as part of the systemic involvement. While pericardial effusion can result from numerous etiologies, including idiopathic causes and infections, particularly viral pathogens such as coxsackievirus and echovirus (55, 56), a small-scale Japanese study identified pericardial involvement in 20% of patients with CAEBV infection (27).

We found 8 articles (21, 22, 24, 27, 39, 42, 43, 47) comprising 14 patients reporting CAEBV-associated pericardial pathologies; 4 were from Japan (7 patients), 2 from China (5 patients), and 1 each from Brazil and India. Of these 14 patients, 10 were pediatric (71.4%) and 4 were adults (28.6%). There were 5 males (35.7%) and 9 females (64.3%). The overall median age was 13 years (range, 3–37 years), with pediatric patients aged 6 years (range, 3–16 years) and adult patients aged 29 years (range: 19–37 years).

Clinical manifestations range from asymptomatic presentation to chest tightness, dyspnea, cardiac tamponade, and even cardiac arrest, depending on the severity of the effusion. CAEBV-related pericardial effusion is usually exudative, with cytological analysis revealing EBV positivity, metagenomic next-generation sequencing demonstrating elevated EBV-DNA levels, and flow cytometry showing predominantly mature T cell populations. Notably, some studies have observed disproportionately high EBV-DNA loads in effusions with minimal cellular components, suggesting that viral replication originates from adjacent tissues rather than the direct infiltration of EBV-infected lymphocytes into the pericardium (24, 57). Echocardiography achieves nearly 100% diagnostic accuracy in detecting pericardial effusion and plays a pivotal role in monitoring and guiding pericardiocentesis. Echocardiography is sufficient for diagnosing pericardial effusion in most patients. However, computed tomography (CT) or magnetic resonance imaging (MRI) may occasionally be used to supplement the evaluation, with CT attenuation values potentially offering additional insights into the composition of the effusion.

### 3.5 Pulmonary arterial hypertension

PAH associated with CAEBV infection is a rare complication characterized by elevated pulmonary artery pressure, and clinically manifests as progressive dyspnea, edema, and right-sided heart failure. Although PAH can occur in both pediatric and adult populations, it is more commonly observed in adults and typically presents as an isolated PAH.

Our review identified 7 articles (28, 29, 31, 34, 35, 38, 45) with 10 patients reporting CAEBV-associated PAH: 6 from Japan (9 patients) and 1 from China. Among the 10 patients, 2 were pediatric (20%) and 8 were adults (80%), with a male-to-female ratio of 4:6 (40% vs. 60%). The overall median age was 32 years (range, 6–69 years), with pediatric cases aged 6–9 years, and the median age of the adult subgroup was 38 years (range, 23–69 years). Both pediatric patients had CALs, whereas among the adults, 7 exhibited isolated PAH and 1 developed heart failure.

The clinical classification of PAH comprises 5 groups, and CAEBV-associated PAH is categorized as group 5, which is characterized by unclear and multifactorial pathogenic mechanisms (58). In all patients with CAEBV-associated PAH, right heart catheterization revealed elevated pulmonary vascular resistance and normal pulmonary capillary wedge pressure, indicative of precapillary pulmonary hypertension. The diagnosis of PAH is relatively straightforward, and echocardiography is the primary tool for evaluating pulmonary artery pressure by measuring the tricuspid regurgitation velocity. Right heart catheterization remains the gold standard for definitive diagnosis. When diagnosing PAH, it is crucial to systematically exclude common etiologies across all PAH groups. Once these causes have been ruled out, rare etiologies such as CAEBV infection should be considered. Conversely, in patients with CAEBV infection and PAH, the aforementioned etiological groups must be excluded before attributing PAH to CAEBV-induced pathogenesis.

### 3.6 Valvular regurgitation

Patients with CAEBV infection may develop valvular dysfunction as the disease progresses, although its precise pathogenesis remains unclear. Potential mechanisms could involve EBV-mediated valvular damage or result from cardiac pathologies such as cardiomyopathy, myocardial infarction, papillary muscle displacement and dysfunction, or arrhythmia-induced valvular abnormalities. In our review, we found that CAEBV-associated valvular regurgitation predominantly affected the mitral, tricuspid, and aortic valves.

Five articles reporting CAEBV-related valvular dysfunction comprising 9 patients were reviewed. There were 3 Chinese studies (37, 39, 41) (7 patients), and 1 study each with 1 patient from Japan (25) and India (47). The cohort included 8 pediatric and 1 adult patients, with a male-to-female ratio of 3:6. The overall median age was 10 years (range, 4–37 years), stratified as 7 years (range, 4–14 years) in the pediatric subgroup and 37 years in the adult subgroup. Aortic regurgitation was observed in 7 patients, mitral regurgitation in 5, and tricuspid regurgitation in 2. No isolated valvular regurgitation was observed. Instead, among the pediatric cases, 7 coexisted with CALs, 1 with aortic sinus dilatation, and the adult case presented with both a ventricular aneurysm and vascular involvement.

Echocardiography is the primary non-invasive diagnostic modality for valvular heart disease, enabling real-time assessment of valve morphology, function, and hemodynamics as well as semiquantitative evaluation of the severity of stenosis and regurgitation. Cardiac magnetic resonance (CMR) can accurately quantify regurgitant volumes and evaluate myocardial fibrosis or scarring. Digital subtraction angiography (DSA) provides precise measurements of transvalvular pressure gradients, cardiac output, and valve orifice area; left ventriculography enables direct visualization of regurgitant severity and evaluation of coronary involvement. CT primarily aids in detecting valvular calcification and excludes coronary artery disease, offering additional anatomical characterization in complex cases.

### 3.7 Myocarditis or cardiomyopathy

CAEBV-associated myocarditis and cardiomyopathy have rarely been reported. Chen et al. reported 7 patients with EBV-related myocarditis, with only 1 definitively associated with CAEBV infection (9). Our review identified one case each of CAEBV-associated myocarditis and dilated cardiomyopathy (23, 26) one each from Japan and Germany, both involving adult males.

The definitive diagnosis of CAEBV-related myocarditis remains challenging, typically necessitating endomyocardial biopsy for viral genome identification through PCR. However, myocardial biopsies are seldom performed in clinical practice because of the risks involved. As a result, diagnosis relies on clinical manifestations and ancillary tests such as cardiac biomarkers, ECG, echocardiography, angiography, and CMR. In CAEBV-associated myocarditis, elevations in troponin I/T, creatine kinase, and CRP levels may occur, although these findings are not specific. ECG may reveal ST-segment deviations and bundle branch blocks, suggesting myocardial injury. Although echocardiographic findings are usually variable and nonspecific, they can assist in excluding alternative etiologies.

CAEBV with myocardial involvement may lead to ventricular aneurysm formation. Raghuram et al. reported a patient with CAEBV presenting with a left ventricular apical pseudoaneurysm accompanied by thrombus formation with no evidence of abnormal coronary arteries. CMR revealed an apical pseudoaneurysm with wall thinning and transmural late gadolinium enhancement, suggesting myocardial fibrosis (47). Angiography and coronary CT angiography play a crucial role in distinguishing these aneurysms from postinfarction variants because aneurysms following infarction are typically confined to territories supplied by occluded coronary arteries (59).

### 3.8 Heart failure

Heart failure is defined as a clinical syndrome resulting from structural or functional cardiac abnormalities that impair left ventricular ejection fraction, leading to reduced cardiac output and elevated intracardiac pressure. It may develop secondary to CAEBV-related complications including CALs, myocarditis, myocardial ischemia, arrhythmias, PAH, or valvular regurgitation. The clinical manifestations include chest tightness, dyspnea, exercise intolerance, and edema. We identified 7 patients with heart failure (23, 26, 27, 29, 38) (4 adults and 3 children; 2 males and 5 females). The overall median age was 28 years (range, 3–45 years), with a median of 11 years (range, 3–16 years) for pediatric patients and 36 years (range, 28–45 years) for adult patients.

Diagnosis of heart failure integrates clinical signs and symptoms with elevated B-type natriuretic (BNP) or N-terminal pro-B-type natriuretic peptide (NT-proBNP) levels, although determining the underlying etiology remains essential. Echocardiography is the primary diagnostic modality and can comprehensively assess cardiac structure and systolic and diastolic function while excluding valvular, cardiomyopathic, ischemic, or congenital etiologies. CMR precisely quantifies left ventricular systolic function and diagnoses cardiomyopathy- or myocarditis-related heart failure. ECG can be used to diagnose arrhythmia and ischemic cardiomyopathy, while DSA is used to evaluate coronary artery disease, which is a potential cause of heart failure. Invasive hemodynamic monitoring is indicated for patients with acute decompensated heart failure and suspected cardiogenic shock, providing a precise assessment of volume status and cardiac function.

## 4 Imaging features

Echocardiography is the primary imaging modality for assessing cardiac structure and function. This test should be routinely performed when cardiac involvement related to a CAEBV infection is suspected. On echocardiography, CALs often manifest as dilation or widening of the left and right coronary arteries, potentially progressing to CAA formation, with some patients developing thrombosis (37). Some patients may exhibit left ventricular enlargement accompanied by systolic dysfunction, and may even develop regional wall motion abnormalities or ventricular aneurysms. Valvular involvement typically manifests as valvular regurgitation. Pulmonary hypertension, characterized by right heart enlargement and pulmonary artery dilatation, can also be observed. Pericardial effusion can occur in some patients, necessitating pericardiocentesis and drainage, when indicated.

CT angiography provides detailed visualization of cardiac and vascular anatomy. It clearly delineates the specific morphology, size, and location of the coronary artery aneurysms. Furthermore, CT angiography can detect aortic involvement such as aortic wall thickening, luminal stenosis, or dilation. DSA is a routine diagnostic procedure. However, in patients with suspected multiple medium-sized or giant coronary artery aneurysms, coronary angiography is recommended to evaluate the extent of CALs. Coronary angiography offers precise delineation of the coronary anatomy and morphology, accurate determination of the degree of coronary dilation or stenosis, and identification of thrombosis. However, its invasive nature limits its clinical application.

CMR offers unique advantages in characterizing myocardial tissue properties, enabling non-invasive detection of myocardial inflammation and fibrosis. T2-weighted imaging can detect myocardial edema, which is indicative of active inflammation. Late gadolinium enhancement patterns can be used to identify myocardial fibrotic regions. In CAEBV-associated myocarditis, this may present as a subepicardial or patchy enhancement. T1 mapping and extracellular volume fraction, which are quantitative parameters, allow for earlier and more objective detection of diffuse myocardial interstitial abnormalities. CMR also clearly depicts pericardial thickening, effusion, and abnormal signals in the walls of major vessels (33).

Positron emission tomography (PET)-CT evaluates disease activity at the metabolic level, providing substantial value in determining the extent of inflammation and guiding treatment. Fludeoxyglucose F18 (FDG) PET may reveal focal or diffuse increased myocardial metabolic activity, suggesting active inflammation. When CAEBV involves the vasculature, PET-CT can detect abnormally increased FDG uptake in the walls of the large arteries, indicating large-vessel vasculitis. Metabolic parameters derived from PET-CT can serve as objective indicators for assessing responses to treatments such as hematopoietic stem cell transplantation (37).

## 5 Treatment and prognosis

No standardized therapeutic regimen exists globally for CAEBV infections. Antiviral monotherapy demonstrates limited efficacy, whereas immunosuppressive agents, cytotoxic chemotherapy, and cellular therapies provide only transient responses, leading to most patients eventually experiencing relapse or disease progression (60–62). Allogeneic hematopoietic stem cell transplantation (HSCT) is generally considered the only potentially curative option for CAEBV; however, the supporting survival estimates are derived from retrospective/observational studies and should be interpreted in context (study population, setting, and potential selection bias). For example, a Japanese cohort study reported a 15-year overall survival rate of 60.6% in transplanted patients compared with 25.7% in non-transplanted patients (63, 64).

Management of CAEBV-associated cardiovascular complications remains challenging, requiring guideline-directed concurrent treatment of both primary infections and secondary pathologies. Antiplatelet and anticoagulation therapies constitute the therapeutic cornerstone of CALs, and long-term regimens are recommended for patients experiencing thrombotic or embolic events (49). CABG has demonstrated benefits in patients with CAL and chronic coronary occlusion-induced myocardial infarction (46). Anticoagulation therapy combined with revascularization may be considered for patients with multivessel involvement. The management of pericardial effusion relies on echocardiographic quantification, with pericardiocentesis indicated for patients with hemodynamic instability. Treatment of PAH lacks a consensus, but may include PAH-specific vasodilators, such as endothelin receptor antagonists and phosphodiesterase-5 inhibitors, which have been reported to transiently improve hemodynamics in some patients (29, 35, 45). Malignant arrhythmias may require anti-arrhythmic therapy, defibrillation, or temporary pacemaker implantation. Valvular regurgitation management is guided by risk stratification, although no surgical interventions were reported due to mild-to-moderate regurgitation (65). Myocarditis requires vigilant cardiac monitoring and supportive care, while heart failure requires aggressive pharmacotherapy with inotropes, vasodilators, and diuretics (66, 67).

Cardiovascular involvement in patients with CAEBV infection is associated with a poor prognosis, as evidenced by the overall mortality rate of approximately 53% in our analysis. This correlates with the EBV load, severity of cardiovascular injury, and extent of multiorgan involvement. Patients with pericardial effusion and PAH demonstrated relatively favorable outcomes, with resolution of the effusion and reduced pulmonary pressure after treatment. Notably, 2 patients with PAH achieved successful pregnancies (38). Ethnic disparities in prognosis are evident as Western patients exhibit better survival rates than their Asian counterparts. This may be linked to divergent cell lineage involvement, with T or NK-cell predominance observed in Asian populations and B-cell proliferation being more common in Western patients (68). Clinicians should increase awareness of CAEBV-associated cardiovascular complications to facilitate early recognition, accurate risk stratification, and tailored management strategies.

## 6 Limitations of the evidence

Our study has certain limitations that should be acknowledged. The conclusions drawn from this review are constrained by the nature of the available evidence, which predominantly consisted of case reports and small case series. This body of literature is susceptible to publication bias, where severe or unusual presentations are more likely to be reported, and selection bias, as most reported cases originated in East Asia. The retrospective and heterogeneous design of these studies limits the standardization of diagnostic criteria, treatments, and outcome assessments. Furthermore, the absence of large-scale prospective or controlled studies hinders the establishment of causality and robust evaluation of therapeutic strategies. Future collaborative multicenter studies are essential to generate higher-level evidence for the management of cardiovascular complications in CAEBV infection.

## 7 Conclusion

CAEBV infection is associated with a broad spectrum of cardiovascular manifestations, including CALs, multivessel aneurysms, pericardial effusion, PAH, arrhythmias, valvular dysfunction, myocarditis, and heart failure and presents diagnostic and therapeutic challenges. The systematic evaluation of cardiovascular involvement is crucial for timely intervention and long-term surveillance. The poor prognosis of CAEBV-associated cardiovascular complications highlights the urgent need for further research to elucidate its pathogenic mechanisms and optimize therapeutic strategies.
