# Supplementary material for: Cardiovascular complications in chronic active Epstein-Barr virus infection: a narrative review
Source: Front Cardiovasc Med. 2026 Mar 18;13:1651391. doi: 10.3389/fcvm.2026.1651391 (PMC13038964; doi:10.3389/fcvm.2026.1651391)
Supplement: Supplementary file 1 [file Table1.docx]

Supplementary Table. Therapeutic Regimens and Outcomes in CAEBV Patients with Cardiovascular Complications.

| Year | Author | Country | Patient | Therapeutic regimens | Outcomes |
| --- | --- | --- | --- | --- | --- |
| 1989 | Kobayushi et al.^20^ | Japan | Pt.1 | antivirals, immunosuppressants, chemotherapy | NA |
| 1992 | Yamada et al.^21^ | Japan | Pt.2 | steroids, antivirals, immunosuppressants | died of respiratory failure |
| 1993 | Kikuta et al.^22^ | Japan | Pt.3 | NA | death (unknown cause) |
|  |  |  | Pt.4 | NA | death (unknown cause) |
|  |  |  | Pt.5 | NA | death (unknown cause) |
| 1996 | Nakagawa et al.^11^ | Japan | Pt.6 | antivirals, immunosuppressants | died of respiratory failure |
| 1998 | Murakami et al.^12^ | Japan | Pt.7 | steroids, immunosuppressants | died of respiratory failure |
| 2001 | Hauptmann et al.^23^ | Germany | Pt.8 | steroids, antivirals, chemotherapy | died of heart failure |
| 2006 | Toubo et al.^24^ | Japan | Pt.9 | steroids, antivirals | death (unknown cause) |
| 2006 | Sato et al.^25^ | Japan | Pt.10 | chemotherapy | death (unknown cause) |
| 2008 | Takano et al.^26^ | Japan | Pt.11 | steroids, immunosuppressants, chemotherapy | died of multiple organ failure and disseminated intravascular coagulation |
| 2009 | Muneuchi et al.^27^ | Japan | Pt.12 | steroids, immunosuppressants | died of transplantation-related event |
|  |  |  | Pt.13 | steroids | alive |

Supplementary Table. (Continued)

| Year | Author | Country | Patient | Cardiac complications | Outcomes |
| --- | --- | --- | --- | --- | --- |
| 2009 | Muneuchi et al.^27^ | Japan | Pt.14 | steroids, immunosuppressants | died of transplantation-related event |
|  |  |  | Pt.15 | steroids, antivirals, chemotherapy | died of multiple organ failure |
|  |  |  | Pt.16 | steroids, antivirals, chemotherapy | alive |
|  |  |  | Pt.17 | steroids, immunosuppressants | died of multiple organ failure |
|  |  |  | Pt.18 | steroids, immunosuppressants, chemotherapy | sudden death |
|  |  |  | Pt.19 | steroids, chemotherapy | died of multiple organ failure |
|  |  |  | Pt.20 | steroids, chemotherapy | died of transplantation-related event |
| 2009 | Hasegawa et al.^10^ | Japan | Pt.21 | allo-HSCT | died of sudden circulatory failure |
| 2011 | Hashimoto et al.^28^ | Japan | Pt.22 | steroids, immunosuppressants, chemotherapy | died of circulatory failure |
| 2014 | Onishi et al.^29^ | Japan | Pt.23 | allo-HSCT | alive |
| 2014 | Nishimura et al.^30^ | Japan | Pt.24 | allo-HSCT | NA |
| 2015 | Fukuda et al.^31^ | Japan | Pt.25 | allo-HSCT | died of septic shock and multiple organ failure |
| 2015 | Kim et al.^32^ | Korea | Pt.26 | steroids, antivirals, immunosuppressants, chemotherapy, allo-HSCT | died of lymphoma |

Supplementary Table. (Continued)

| Year | Author | Country | Patient | Cardiac complications | Outcomes |
| --- | --- | --- | --- | --- | --- |
| 2016 | Jiang et al.^33^ | China | Pt.27 | steroids, immunosuppressants | NA |
| 2019 | Ba et al.^34^ | China | Pt.28 | steroids, chemotherapy | alive |
| 2020 | Akagi et al.^35^ | Japan | Pt.29 | chemotherapy, allo-HSCT | died of septic shock and multiple organ failure |
| 2020 | Kang et al.^36^ | Japan | Pt.30 | steroids | alive |
| 2020 | Xiao et al.^37^ | China | Pt.31 | allo-HSCT | alive |
| 2021 | Sasagasako et al.^38^ | Japan | Pt.32 | allo-HSCT | alive |
|  |  |  | Pt.33 | allo-HSCT, | alive |
| 2021 | Wei et al.^39^ | China | Pt.34 | chemotherapy | alive |
|  |  |  | Pt.35 | refusal | alive |
|  |  |  | Pt.36 | refusal | alive |
|  |  |  | Pt.37 | refusal | alive |
|  |  |  | Pt.38 | chemotherapy | alive |
|  |  |  | Pt.39 | chemotherapy, allo-HSCT | alive |

Supplementary Table. (Continued)

| Year | Author | Country | Patient | Cardiac complications | Outcomes |
| --- | --- | --- | --- | --- | --- |
| 2021 | Wei et al.^39^ | China | Pt.40 | chemotherapy, allo-HSCT | death (unknown cause) |
|  |  |  | Pt.41 | chemotherapy, allo-HSCT | alive |
|  |  |  | Pt.42 | chemotherapy | alive |
|  |  |  | Pt.43 | chemotherapy, allo-HSCT | alive |
| 2021 | Jamal et al.^40^ | Morocco | Pt.44 | chemotherapy | death (unknown cause) |
| 2022 | Li et al.^41^ | China | Pt.45 | antivirals | death (unknown cause) |
| 2022 | Pi et al.^42^ | China | Pt.46 | immunosuppressants | alive |
| 2022 | Paula et al.^43^ | Brazil | Pt.47 | NA | death (unknown cause) |
| 2022 | Teng et al.^43,44^ | China | Pt.48 | immunosuppressants | alive |
| 2023 | Misaki et al.^45^ | Japan | Pt.49 | allo-HSCT | alive |
|  |  |  | Pt.50 | chemotherapy | died of respiratory failure |
|  |  |  | Pt.51 | steroids, chemotherapy | died of lymphoma progression. |

Supplementary Table. (Continued)

| Year | Author | Country | Patient | Cardiac complications | Outcomes |
| --- | --- | --- | --- | --- | --- |
| 2023 | Misaki et al.^45^ | Japan | Pt.52 | allo-HSCT | died of respiratory and circulatory failure |
| 2023 | Qian et al.^19^ | China | Pt.53 | antivirals, chemotherapy, allo-HSCT | alive |
|  |  |  | Pt.54 | antivirals, allo-HSCT | alive |
|  |  |  | Pt.55 | antivirals, chemotherapy, allo-HSCT | alive |
| 2024 | Iwata et al.^46^ | Japan | Pt.56 | allo-HSCT | alive |
| 2024 | Raghuram et al.^47^ | India | Pt.57 | steroids, immunosuppressants | died of sudden cardiac arrest |

HSCT, hematopoietic stem cell transplantation; NA, not available/not reported; 'refusal' indicates refusal of further intensive therapy (e.g., chemotherapy/HSCT) as described in the original report.
